# Treatment of tegumentary leishmaniasis in two hemodialysis patients with end-stage renal disease using two series of pentamidine

**DOI:** 10.1590/0037-8682-0633-2020

**Published:** 2021-03-22

**Authors:** Karla Cristina Petruccelli, Kátia Nascimento Couceiro, Maria das Graças Vale Barbosa Guerra, Zanair Soares Vasconcelos, Alba Regina Jorge Brandão, Monica Hosannah Silva e Silva, Jorge Augusto de Oliveira Guerra

**Affiliations:** 1 Universidade do Estado do Amazonas, Programa de Pós-Graduação Stricto Sensu em Medicina Tropical, Manaus, AM, Brasil.; 2 Universidade Federal do Amazonas, Hospital Universitário “Getúlio Vargas”, Manaus, AM, Brasil.; 3 Universidade do Estado do Amazonas, Manaus, AM, Brasil.; 4 Fundação de Medicina Tropical “Heitor Vieira Dourado”, Manaus, AM, Brasil.; 5 Exército Brasileiro, Hospital Militar de Área de Manaus, Manaus, AM, Brasil.; 6 Faculdade Metropolitana de Manaus, Manaus, AM, Brasil.

**Keywords:** Leishmania, Pentamidine, End-stage renal disease

## Abstract

In this study, we present two cases of cutaneous leishmaniasis in patients with end-stage renal disease, who were treated solely with intramuscular pentamidine. In such cases, treatment implies a fine line between therapeutic efficacy and toxicity. This is suggestive of a knowledge gap; however, findings indicate that this is still the fastest and safest alternative to the treatment with antimonials. Also, it can help avoid the side effects that occur upon using antimonials.

## INTRODUCTION

American tegumentary leishmaniasis (ATL) is a zoonotic disease that is transmitted through sandflies (Diptera: Psychodidae) and caused by several species of *Leishmania* parasite (Kinetoplastida: Trypanosomatidae). It is considered as a neglected infectious disease occurring in tropical regions, and its epidemiology is complex with intra- and inter-specific variations in transmission cycles, clinical manifestations, and variable responses to therapy^1^.

There are few studies that demonstrated the treatment of ATL with pentamidine in patients with renal impairment. To our knowledge, there are only two reports of such cases. The first very recent report describes a patient with refractory mucousal leishmaniasis (RML) and chronic kidney disease (CKD). In this case, RML exhibited recurrence after the treatment with amphotericin B, and was then treated with pentamidine. However, the patient presented with hyperglycemia and temporary worsening of renal complications, though eventually exhibited a complete cure of ATL^2^. The second case report describes a patient with visceral leishmaniasis (VL) and HIV, who presented with end-stage renal disease (ESRD) and had a perpetual need for dialysis due to the administration of miltefosine. This patient’s treatment was changed to pentamidine and fluconazole, and thus, exhibited a complete cure of ATL^3^. However, there are no studies relating the pharmacokinetics of pentamidine with the treatment of ATL in dialysis patients^4^.

Pentavalent antimonials are the medication of choice for the treatment of cutaneous leishmaniasis. If there are renal, cardiac, or hepatic comorbidities, or if the patient is pregnant or over 50 years of age, the medication of choice is liposomal amphotericin B, while pentamidine and amphotericin B deoxycholate are considered as the second-line drugs^5^. Pentamidine is often implicated in causing diabetes and kidney damage^6^ ^,^ ^7^. In the same vein, several side effects, including death, are attributed to the use of antimonials and amphotericin^5^. At the Tropical Medicine Foundation Heitor Vieira Dourado (FMT-HVD) in the Amazonas State, Brazil, an average of 1,000 cases of ATL are treated per year; however, 30% of these patients are treated with pentamidine as the first-line drug. All patients undergo renal and hepatic assessments before and after the treatment. Renal alterations have not been reported, and hepatic and/or hematological involvement associated with this medication is rare^8^. Furthermore, in cutaneous or mucocutaneous leishmaniasis, which is caused by several species of *Leishmania,* renal manifestations related to the parasite have not been reported, and this event is usually associated with the choice of medication used for the treatment^5^ ^,^ ^9^. For this reason, pentamidine was considered as the most viable therapeutic alternative for the two ATL patients presented with ESRD.

Herein, we present two cases of ATL in patients with ESRD, both undergoing hemodialysis, in order to report the safety and effective therapeutic response that resulted upon using pentamidine. We highlight the relevance of this case report as it is one of the few pharmacokinetic studies in which the individualization of ATL treatment and the importance of pentamidine as the best option for the treatment of ATL in patients with ESRD have been reported^9^ ^-^ ^11^. To our knowledge, till date, there have been no other case reports similar to the two reported herein.

## CASE REPORT

**FIGURE 1:**
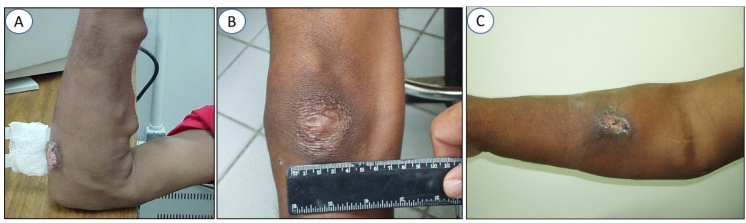
Features of lesions in two cases of American cutaneous leishmaniasis in hemodialysis patients with ESRD. **(A)** Upper limb lesion, 4 cm in diameter. **(B)** Healed upper limb lesion after six months of treatment. **(C)** Ulcerated upper limb lesion, 3 cm in diameter.

**Patient 1:** A 40-year-old male patient with ESRD was experiencing secondary chronic hypertension. The patient routinely took propranolol and minoxidil for his heart issues. At the time of treatment, he was on dialysis for the last eight years. He arrived at the FMT-HVD presented with three cutaneous ATL lesions, one on an upper limb (UL), and two on the lower limbs (LL). During admission, the lesions had already evolved for 15 days and ranged from 1.5 to 4 cm in diameter (Figure 1A-C). Initially, pentamidine was administered intramuscularly (IM) at a dose of 4 mg/kg, in a series of three sessions with an interval of seven days between each session, and administered on D1, D8, and D15. On D48, lesions were still present and a new series of three sessions was administered (D48, D55, D62). The lesions completely healed by D74. In the first treatment series, an episode of vomiting occurred 2 h after one of the doses of pentamidine, although it was the only adverse effect noted upon the use of this medication. 

In the examinations performed on D28, creatinine was found to be 6.8 mg/dL (normal limit: 0.6-1.3 mg/dL) and post-dialysis urea was 61 mg/dL (normal limits: 15-40 mg/dL). The average level of creatinine and urea post-dialysis, during the 8 years prior to treatment with pentamidine, were 10.63 and 60 mg/dL, respectively. In 2003, after treatment with pentamidine, the levels of creatinine and urea were 9.48 and 67.08 mg/dL, respectively. The results of the examinations performed after the second series of treatment for creatinine and urea were 9 and 126 mg/dL, respectively. Mean creatinine level was stable at 9.48 mg/dL. Complete blood count, glycemia, GOT (glutamic oxalacetic transaminase enzyme), TGP (glutamic-pyruvic transaminase enzyme) and amylase did not exhibit any alterations.

The patient was monitored for six months regularly until all the cutaneous lesions healed (Figure 1B). He is still on hemodialysis and exhibits similar laboratory results for creatinine and urea compared to the time before the administration of pentamidine.

**Patient 2:** A 60-year-old male with end-stage renal disease presented at FMT-HVD after a snakebite. He had been on dialysis for five years at the time of initiation of the treatment, and had an ulcerated lesion, which was 3 cm in diameter that erupted 30 days before he attended the leishmaniasis clinic (Figure 1C). This patient was treated with pentamidine at a dose of 4 mg/kg (IM) in three series with seven-day intervals. Doses were administered on days D1, D8, and D15. No side effects were reported. In the examinations performed on D14, the levels of creatinine and post-dialysis were 11.8 and 69 mg/dL, respectively. Except amylase (122 U/L; normal: 0-95 U/L), complete blood count (CBC), glycemia, GOT, and TGP did not exhibit any alterations. During 2004, the mean levels of creatinine and post-dialysis urea were 12.44 and 64 mg/dL, respectively. These values are considered to be within the usual range for patients undergoing dialysis. After six months of follow-up, the lesions healed completely.

## DISCUSSION

Clinical data on the pharmacokinetics of drugs in patients with ESRD are still relatively limited. Therefore, the current recommended doses are considered only as a corollary, and adjustments through therapeutic monitoring and clinical judgment are required to be implemented^12^. In this regard, despite the potential toxicities of pentamidine^5^ ^,^ ^9^ and its large-scale use at the FMT-HVD, evidence of kidney injury or rhabdomyolysis has never been associated with the administration of pentamidine.

Bacterial infections in patients with ESRD, which are considered to be the main reason of morbidity and mortality in this condition^12^, require antibiotic dose balance during the therapy to eliminate the pathogens, prevent bacterial resistance, and alleviate toxicity. Pentamidine interferes with the synthesis of nucleic acids and promotes a leishmanistatic effect that depends on minimal inhibitory concentrations (MIC) for its effectiveness^5^. Therefore, the ideal adjustment is required for the time-dependent use of antibiotics. In this case, smaller doses are recommended at the same frequency as the usual dose in order to guarantee MIC without inducing toxic effects^12^.

The safety levels of the doses, such as those used in the treatment of ATL at the FMT-HVD, for non-dialysis patients, and the implications of higher doses than those recommended in relation to renal, hepatic, or medullary toxicity have been widely investigated^5^ ^,^ ^8^ ^,^ ^9^. However, there are still no reports on the pharmacokinetics and MIC levels of pentamidine tested in patients with leishmaniasis^4^, nor are there any studies involving patients with both ESRD and leishmaniasis.

In one of the cases described in this report, despite the cumulative potential of pentamidine^5^, it was necessary to repeat the treatment, and complete healing of the lesions occurred only after administering the second series of treatment. This result, which is associated with the lack of data on MIC levels for patients with leishmaniasis and the large percentage of recurrences^1^ recorded in the treatment of ATL, indicates a gap in the knowledge thereby raising the question whether the therapeutic failures are inevitable or just need an adjustment of the dose to meet the pharmacokinetic characteristics of individual patient.

The importance of an adequate balance between therapeutic effectiveness and toxicity is evident when analyzing the clinical manifestations among the patients in this report. Despite the use of the same therapeutic regimen, its efficiency and toxicity were notably different. Patient 1, who experienced an episode of vomiting as a mild side effect in one of the first series of treatment unexpectedly required a second series of treatment, which indicates that pentamidine did not reach to an adequate level of plasma concentration, or that it did not remain in the system for an adequate duration to eliminate the parasite. However, Patient 2 needed only one series of pentamidine treatment and exhibited no such side effects other than an increase in the level of amylase to 122 U/L (normal: 0-95 U/L). This indicates that the concentration of pentamidine in the plasma was sufficient to guarantee the elimination of the parasite; however, it also caused pancreatic lesions. In both patients, the mean levels of creatinine and urea, the other parameters indicative of toxicity and renal deterioration, remained constant, and did not indicate a need of more frequent hemodialysis.

We therefore conclude that, despite using pentamidine, there were no alterations in the pre-existing condition of chronic renal failure. Additionally, in four reviews on the use of pentamidine in patients with renal impairment, there was a lack of data on the pharmacokinetics of pentamidine used for the treatment of ATL in these hemodialysis patients^3^. The analysis of these two patients with ESRD is clinically important and relevant as it indicates the knowledge gaps present in the therapeutic approach when using pentamidine in such cases. While the renal clearance of pentamidine is only 2% as compared to its plasma clearance, the other medications, including antimonials and amphotericin B, are eliminated mainly via the kidneys, which in the present cases would have increased the likelihood of an overdose and possibly lead to fatal toxic events. 
